# Usability, acceptability, and feasibility of a remote monitoring system for rural-dwelling African-Americans

**DOI:** 10.1093/geroni/igaf122.3443

**Published:** 2025-12-31

**Authors:** Otis Owens, Zachary Beattie, Nicole Sharma, Don Campbell, Thomas Riley, Jon Yeargers, Larry Frye, Jeffrey Kaye

**Affiliations:** University of South Carolina, Columbia, South Carolina, United States; Oregon Health & Science University, Portland, Oregon, United States; Oregon Health & Science University, Portland, Oregon, United States; Life Analytics Incorporated, Woodside, California, United States; Oregon Health & Science University, Portland, Oregon, United States; Life Analytics Incorporated, Woodside, California, United States; Life Analytics Incorporated, Woodside, California, United States; Oregon Health & Science University, Portland, Oregon, United States

## Abstract

Background and

**Purpose:**

This study examined the usability, acceptability, and feasibility of deploying a remote monitoring system in the homes of cognitively healthy rural-dwelling African Americans.

**Methods:**

Ten homes in a southeastern state were outfitted with seven sensors/smart devices for up to three months. A ‘one-time’ survey was distributed online to collect demographic and technology use data. A ‘weekly’ online survey was also distributed to assess changes in health and home occupancy. Thirty-minute interviews were conducted at the study’s conclusion. Descriptive statistics were calculated for quantitative data. Qualitative data was analyzed using thematic analysis. Feasibility was determined by the percentage of days data were collected across all sensors.

**Results:**

Participants, on average, were 72 years old, married, had a college or higher education, and owned a smartphone. Themes emerging from interviews related to technology-use satisfaction, ease of technology use, barriers to technology use, openness to remote monitoring, perceived usefulness of remote monitoring for others, and data usefulness. Overall, users were satisfied with the system, especially its passive nature and ease of use. Some, but few, barriers existed to system use (e.g., remembering to weigh). Participants were open to continuous remote monitoring if their health declined. All participants thought receiving sensor data routinely would support their health maintenance. Suggestions for system improvement will be presented. Data across sensors was continuously collected over half of the days installed.

**Implications:**

Remote monitoring may be culturally-appropriate for empowering rural-dwelling African Americans to age-in-place, but these systems should be easy-to-use and designed with community input.

